# Physiological and Transcriptomic Analysis of Shading Stress Responses in *Calamus viminalis* Seedlings

**DOI:** 10.3390/ijms27177804

**Published:** 2026-08-31

**Authors:** Benxue Chen, Qiang Wu, Yuanyuan Du, Xiao Wei, Yanbing Li, Guanglu Liu

**Affiliations:** 1 College of Design, Zhoukou Normal University, Zhoukou 466001, China; cbx@zknu.edu.cn (B.C.); dy202771@126.com (Y.D.); ww1358168@126.com (X.W.); 2 Henan Key Laboratory of Crop Molecular Breeding and Bioreactor, Zhoukou 466001, China; 3 Zhengzhou Academy of Agricultural Science and Technology, Zhengzhou 450007, China; 15296554146@163.com; 4 Key Laboratory for Bamboo and Rattan, International Center for Bamboo and Rattan, Beijing 100102, China

**Keywords:** *Calamus viminalis*, growth and development, photosynthesis, shading stress, transcriptome

## Abstract

Light critically limits rattan seedling establishment. To elucidate adaptive mechanisms of *Calamus viminalis* under varied light, we conducted a 360-day pot experiment with four shading levels (0%, 20%, 50%, 75%), integrating morphological, physiological, antioxidant, and RNA-seq analyses. Moderate shading (20%) maximized seedling height (46.97 cm) and ground diameter (12.46 mm), increased net photosynthetic rate by 57.92% versus full light, and raised soluble protein/sugar while minimizing MDA and proline—indicating the lowest oxidative damage. Severe shading (75%) suppressed carbon fixation, reduced antioxidant enzymes, aggravated lipid peroxidation, and drastically lowered survival. Transcriptomics revealed intensity-dependent reprogramming: moderate shading activated phenylpropanoid/flavonoid defense pathways; severe shading upregulated photosynthesis/ribosomal genes but constrained overall carbon flux. Four core pathways (photosynthesis, hormone signaling, phenylpropanoid metabolism, carbon metabolism) coordinated shade responses—BR signaling upregulated (shade avoidance) while ABA downregulated under low light; light deprivation globally downregulated LHCB1 and rbcL, impairing capture/assimilation. We identify 20% shading as optimal for *C. viminalis* cultivation and reveal multi-level phenotype–physiology–molecule mechanisms, providing a theoretical basis for *Calamus viminalis* seedling nursery management under controlled nursery conditions.

## 1. Introduction

*Calamus viminalis* (also known as Mengpeng rattan) is a climbing, clustering rattan species belonging to the family *Arecaceae* and genus Calamus [1,2]. It is a valuable renewable non-timber forest resource in tropical and south subtropical rainforests. The edible shoots and high-quality stems used for weaving give this species significant developmental potential and cultivation value [3]. However, due to environmental changes and anthropogenic activities, the distribution range and population size of *C. viminalis* have been declining, and the lack of effective management and cultivation techniques has exacerbated the shortage of rattan resources [4]. The lack of standardized seedling breeding technology restricts the artificial expansion and natural restoration of rattan forest, so it is urgently necessary to carry out targeted cultivation research of seedlings [5].

Light is a fundamental environmental factor influencing plant growth and development, as its intensity and quality regulate photosynthesis, growth patterns, and metabolic processes [6,7,8]. In addition, light, as an important primary regulatory factor, controls various processes such as plant seed germination, leaf expansion, stomatal development, cell division, and photosynthesis [9,10]. In natural ecosystems, the light environment under forest canopies is highly heterogeneous, and seedlings must adapt to varying light conditions for successful establishment [11]. For rattan species, which are climbing palms that rely on surrounding vegetation for support, light availability during the seedling stage is particularly critical [12,13]. Excessive sunlight or heavy shade from overstory vegetation can both result in slow growth, abnormal development, or even mortality [14,15].

Previous studies on various rattan species have revealed species-specific optimal light requirements. For example, *Calamus dioicus* prefers 50–65% relative light, *Calamus simplicifolius* grows best at 20–35% relative light, and *Daemonorops jenkinsiana* and *Calamus rhabdocladus* require approximately 80% relative light intensity for optimal growth [16]. Excessive canopy closure inhibits tillering and stem elongation, prolonging the seedling stage [17]. These findings indicate that moderate shading generally benefits rattan seedling regeneration, though optimal light conditions vary considerably among species.

At the molecular level, plants respond to shading stress by regulating complex gene regulatory networks. For instance, *Arabidopsis thaliana* responds to shade by reducing the inhibitory effect of *BBX24* on the activity of *PIF4* [18]. In tomato [19], the *PIF4* protein responds to light signals and alters the content of carotenoids, thereby influencing fruit ripening. Receptor-like kinases (ER) regulate the content of auxin and gibberellin, thereby influencing the elongation of the hypocotyl and thus responding to shading stress [20]. Collectively, these findings demonstrate that diverse core regulatory proteins and phytohormone signaling cascades synergistically coordinate plant morphological and metabolic adaptation to low-light shade environments at the molecular level.

Despite the recognized importance of light for rattan growth, the molecular mechanisms underlying shade responses in *C. viminalis* remain largely unexplored [5]. Transcriptomics has emerged as a powerful approach for uncovering gene functions and biological mechanisms, particularly in plant stress responses. Recent studies have successfully applied transcriptomic analyses to investigate shade tolerance in various species [21], including foxtail millet [22], *Arabidopsis* [23], and soybean [24]. However, similar investigations in rattan species are scarce.

In this study, we combined physiological measurements and transcriptomic analysis to investigate the responses of *C. viminalis* seedlings to different shading intensities. Our objectives were to: (1) characterize the morphological and physiological changes of *C. viminalis* seedlings under varying light conditions; (2) identify differentially expressed genes and key metabolic pathways involved in shade responses; and (3) uncover physiological and transcriptional shade response rules of container-grown *C. viminalis* seedlings to optimize artificial seedling raising light management. These findings will provide a theoretical basis for *Calamus viminalis* seedling nursery management under controlled nursery conditions.

## 2. Results

### 2.1. Effects of Shading on Growth and Survival of C. viminalis Seedlings

**Figure 1:**
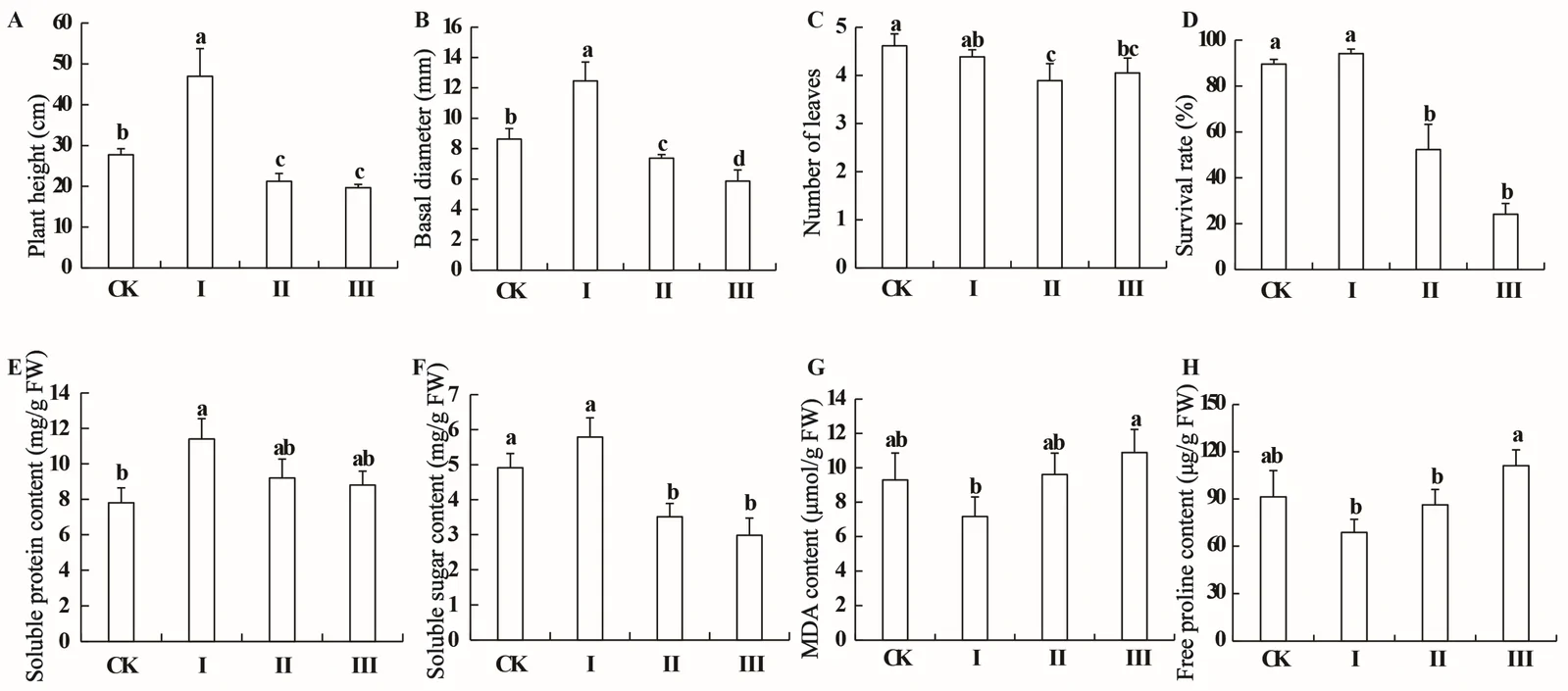
Effects of different shading treatments on growth parameters of Calamus viminalis seedlings. (**A**) Plant height; (**B**) basal diameter; (**C**) leaf number; (**D**) survival rate; (**E**) soluble protein content; (**F**) soluble sugar content; (**G**) MDA content; (**H**) free proline content. CK: 0% shading; T I: 20% shading; T II: 50% shading; T III: 75% shading. Data are presented as mean ± SD (*n* = 30), Different lowercase letters indicate significant differences between groups (*p* < 0.05). There are no significant differences between groups with the same letter.

After 360 days of treatment, different shading intensities significantly affected the growth and survival of *C. viminalis* seedlings. As shown in Figure 1, seedling height and basal diameter exhibited a unimodal response to shading intensity, first increasing and then decreasing with increasing shade. Under Treatment I (20% shading), seedlings achieved maximum height (46.97 cm) and basal diameter (12.46 mm), which were significantly higher than those of the control (full sunlight). In contrast, under Treatment III (75% shading), height, basal diameter, leaf number, and survival rate were all significantly lower than those of the control. Leaf number and seedling survival rate under Treatment I did not differ significantly from the control but were significantly higher than those under Treatment II (50% shading). In terms of osmotic regulation substances, the contents of soluble proteins and soluble sugars in Treatments II and III were significantly lower than those in CK, while there was no significant difference between Treatment I and CK. This indicates that medium and high concentration treatments (II and III) have a significant inhibitory effect on the accumulation of soluble substances. The content of malondialdehyde (MDA) reached the highest level in Treatment III, which was significantly higher than that in CK and Treatment I. The MDA content in Treatment I was the lowest, suggesting that Treatment III may have triggered a strong membrane lipid peroxidation process. The content of free proline was significantly higher in Treatment III than in other treatments, while it was significantly lower in Treatment I and Treatment II than in CK, indicating that the accumulation of proline is only induced under high concentration treatments. These results demonstrate that moderate shading (approximately 20%) promotes the growth and survival of *C. viminalis* seedlings, while excessive shading severely inhibits development and increases mortality.

### 2.2. Effects of Shading on Physiological and Photosynthetic Characteristics

**Figure 2:**
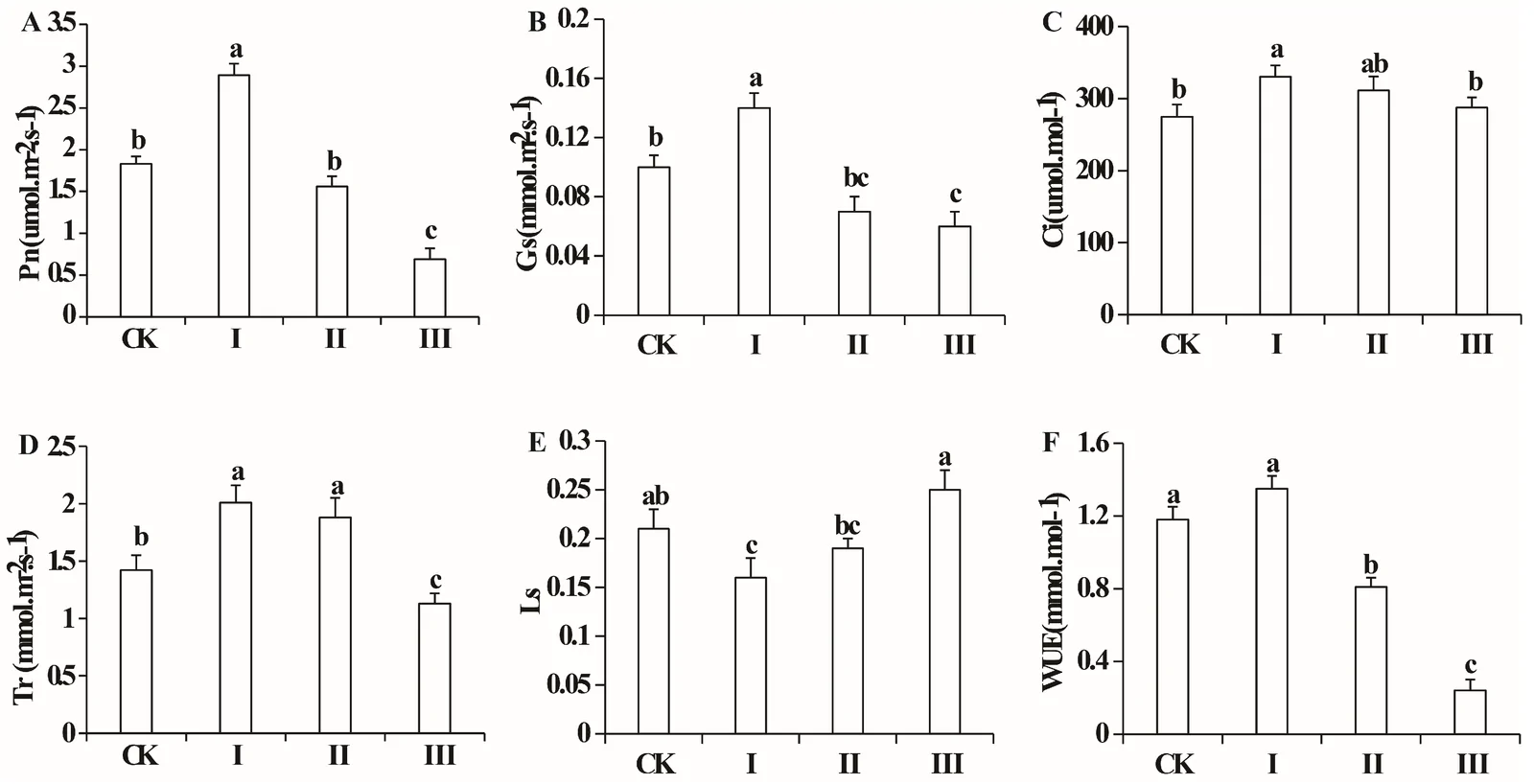
Effects of different shading treatments on photosynthetic parameters of Calamus viminalis seedlings. (**A**) Net photosynthetic rate (Pn); (**B**) stomatal conductance (Gs); (**C**) intercellular CO_2_ concentration (Ci); (**D**) transpiration rate (Tr); (**E**) stomatal limitation value (Ls); (**F**) water use efficiency (WUE). CK: 0% shading; I: 20% shading; II: 50% shading; III: 75% shading. Different letters indicate significant differences at *p* < 0.05. Data are presented as mean ± SD (*n* = 3).

Different shading intensities significantly affected the photosynthetic parameters of *C. viminalis* seedling leaves (Figure 2). The net photosynthetic rate (Pn) exhibited a unimodal response to shading, first increasing and then decreasing with increasing shade intensity. Under Treatment I (20% shading), Pn reached its maximum value, which was significantly higher than that of the control (CK) and Treatment III (75% shading). Similarly, stomatal conductance (Gs) showed the highest value under Treatment I, significantly exceeding CK and Treatment III. Intercellular CO_2_ concentration (Ci) was significantly elevated under Treatment I compared to CK and Treatment III, suggesting enhanced CO_2_ availability for photosynthesis under moderate shading. The transpiration rate (Tr) followed a similar pattern, with the highest value under Treatment I, significantly higher than CK and Treatment III.

In contrast, the stomatal limitation value (Ls) exhibited an opposite trend, with the lowest value under Treatment I, significantly lower than CK and Treatment III. This indicates that stomatal limitation was alleviated under moderate shading, contributing to improved photosynthetic performance. Water use efficiency (WUE) was also significantly affected by shading, with the highest value observed under Treatment I, which was significantly higher than CK and Treatment III, suggesting that moderate shading enhanced both carbon gain and water conservation.

Overall, these results demonstrate that moderate shading (20%) significantly enhances photosynthetic capacity by increasing stomatal conductance, improving CO_2_ availability, and reducing stomatal limitation, while heavy shading (75%) severely suppresses photosynthetic function, leading to reduced carbon assimilation and impaired plant growth.

### 2.3. Transcriptome Sequencing and Differential Expression Analysis 

**Figure 3:**
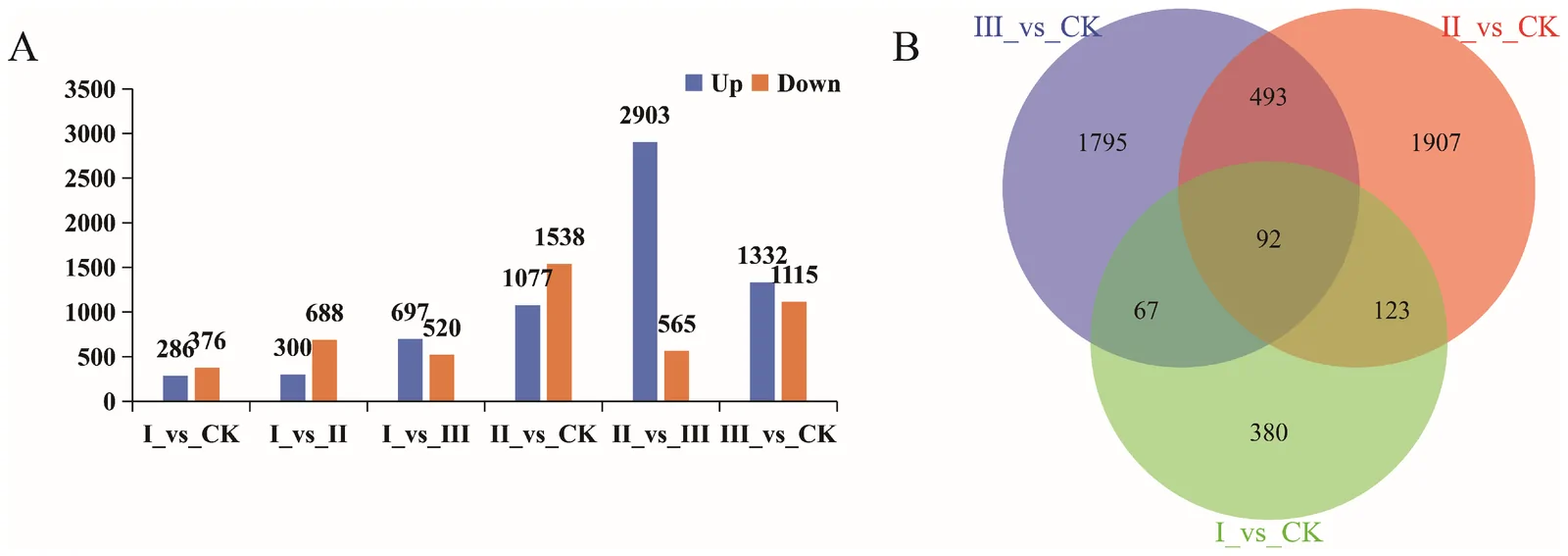
Analysis of unique DEGs in each shading treatment. (**A**) Number of upregulated and downregulated differentially expressed genes (DEGs) in each shading treatment. Blue bars represent upregulated genes, and orange bars represent downregulated genes. (**B**) The Venn diagram comparison of the number of DEGs.

To elucidate the molecular mechanisms underlying shade responses in *C. viminalis* seedlings, RNA-seq analysis was performed on leaf tissues from the four treatment groups (Table S1). After quality filtering and alignment to the reference genome, a total of approximately 21.59 million clean reads were obtained per sample, with an average mapping rate exceeding 85%. Differential expression analysis was conducted by comparing each shading treatment group with the control (CK). As shown in Figure 3A, the number of differentially expressed genes (DEGs) varied considerably among treatments. Under Treatment I (20% shading), a total of 662 DEGs were identified, comprising 286 upregulated genes and 376 downregulated genes. Treatment II (50% shading) exhibited 2615 DEGs, with 1077 upregulated and 1538 downregulated. Notably, Treatment III (75% shading) exhibited 2437 DEGs, including 1332 upregulated and 1115 downregulated genes.

The Venn diagram demonstrates that the transcriptomic reprogramming elicited by Treatment II exhibited the most distinct and abundant DEGs, while treatments II and III shared the largest pool of co-regulated genes. Only a small core set of 92 DEGs was commonly modulated by all three treatments, implying that I, II and III exert both divergent regulatory effects on specific gene subsets and convergent modulation on a conserved transcriptional module.

### 2.4. KEGG Pathway Enrichment Analysis of DEGs

KEGG pathway enrichment analysis revealed distinct functional differentiation among the three shading treatments (Figure S1, Table S2). In Treatment I vs. CK, DEGs were predominantly enriched in phenylpropanoid biosynthesis, flavonoid biosynthesis, tyrosine metabolism, and cytochrome P450 xenobiotic metabolism pathways, indicating that moderate shading primarily activates plant secondary metabolism and detoxification pathways, inducing strong defense and stress responses. In Treatment II vs. CK, DEGs were significantly enriched in phenylpropanoid biosynthesis, flavonoid biosynthesis, starch and sucrose metabolism, and glycolysis/gluconeogenesis pathways. This pattern suggests that moderate-to-heavy shading activates both secondary metabolism and energy metabolism, representing an intermediate phenotype that balances defense and growth. In Treatment III vs. CK, DEGs were highly enriched in photosynthesis, photosynthetic antenna proteins, ribosome, and unsaturated fatty acid biosynthesis pathways. This enrichment pattern indicates that heavy shading significantly promotes photosynthetic efficiency and protein synthesis, manifesting as a growth-promoting and biomass-accumulating phenotype. Notably, the CK group maintained basal metabolic levels without obvious defense activation or growth enhancement.

### 2.5. GO Functional Enrichment Analysis of DEGs

GO functional enrichment analysis revealed that DEGs were broadly distributed across three major categories, namely, Biological Process (BP), Molecular Function (MF), and Cellular Component (CC), with distinct enrichment patterns among treatments (Figure S2).

In Treatment I vs. CK, DEGs showed moderate enrichment across all three categories, primarily associated with stress response, metabolic regulation, and catalytic activity terms, consistent with the defense-oriented regulatory mode of Treatment I. In Treatment II vs. CK, DEGs were predominantly concentrated in Biological Process and Molecular Function categories, mainly involved in energy metabolism, secondary metabolic processes, and oxidoreductase activity regulation, reflecting the intermediate phenotype that balances defense and energy metabolism. In Treatment III vs. CK, DEGs exhibited the highest number and strongest enrichment across all three categories, particularly in Cellular Component terms (chloroplast, thylakoid, ribosome), Biological Process terms (photosynthesis-related, biosynthesis, cellular metabolism), and Molecular Function terms (binding, catalytic, structural molecule activity). This pattern aligns with the growth-promoting and photosynthetic-enhancing phenotype of Treatment III.

### 2.6. Photosynthesis Pathway Gene Expression Under Shading Stress

**Figure 4:**
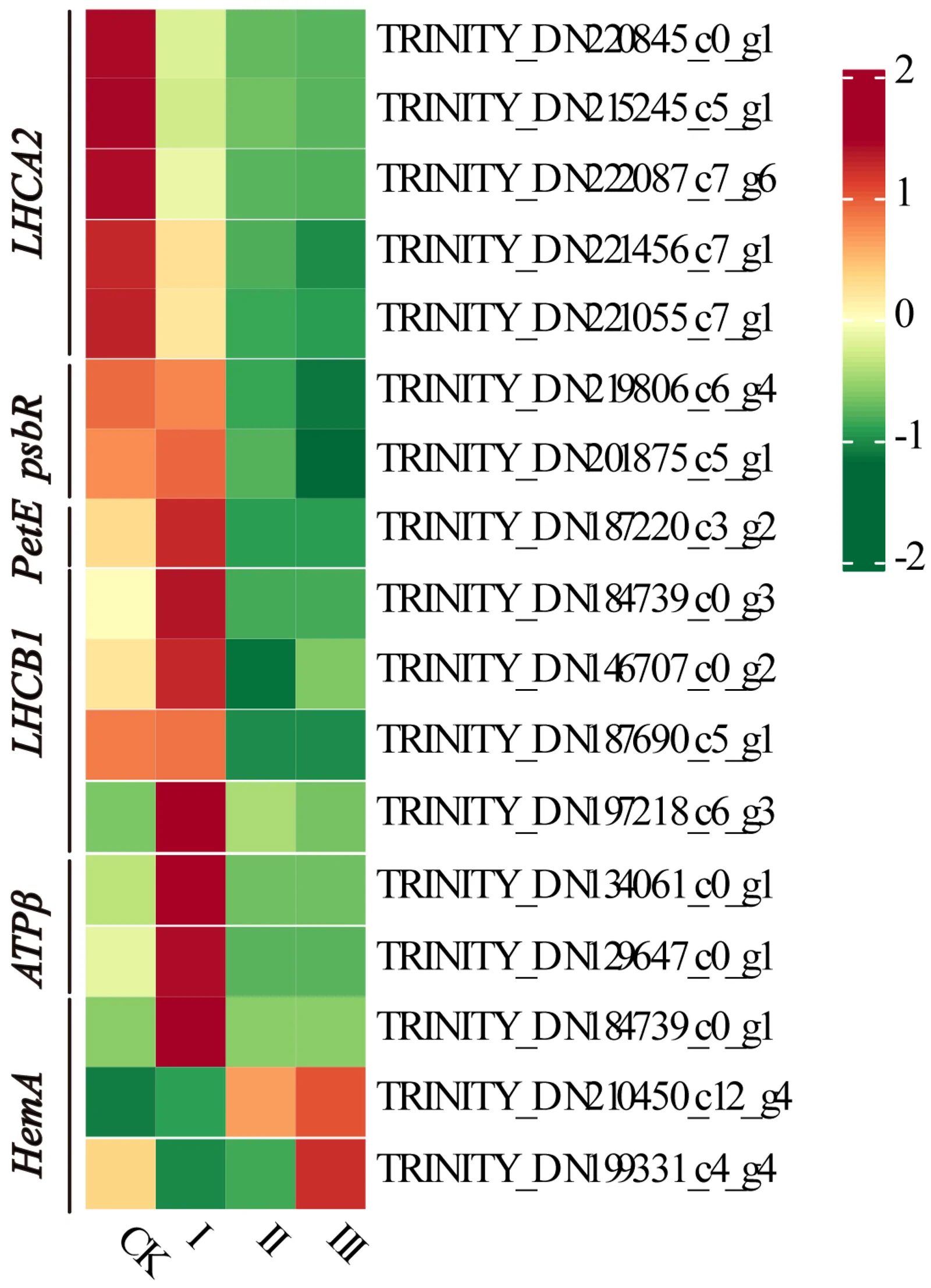
Heatmap illustrating expression profiles of photosynthesis-related Trinity unigenes. Color intensity reflects relative transcript abundance. Key photosynthesis functional gene annotations are marked. Each column represents the mean expression value (log2 FPKM) of three biological replicates obtained from RNA-seq data.

Analysis of photosynthesis pathway genes revealed that shading stress globally suppressed the transcription of genes involved in light harvesting, electron transport, and carbon fixation (Figure 4). Key functional genes, including the light-harvesting chlorophyll protein gene LHCA2, photosystem II core gene psbR, LHCB1, plastocyanin gene PetE, ATP synthase beta subunit ATPβ, and the chlorophyll synthesis key gene HemA, all exhibited negative Z-scores under shading treatments. All Trinity-assembled unigenes (e.g., TRINITY_DN220845_c0_g1) in this pathway showed consistently negative Z-scores across shading samples. These results demonstrate that reduced light intensity globally suppresses the transcription of genes involved in light capture, electron transfer, and light energy conversion, directly reducing light capture and carbon fixation efficiency, representing the molecular basis for decreased photosynthetic rates under shading.

### 2.7. Plant Hormone Signal Transduction Pathway Under Shading Stress

**Figure 5:**
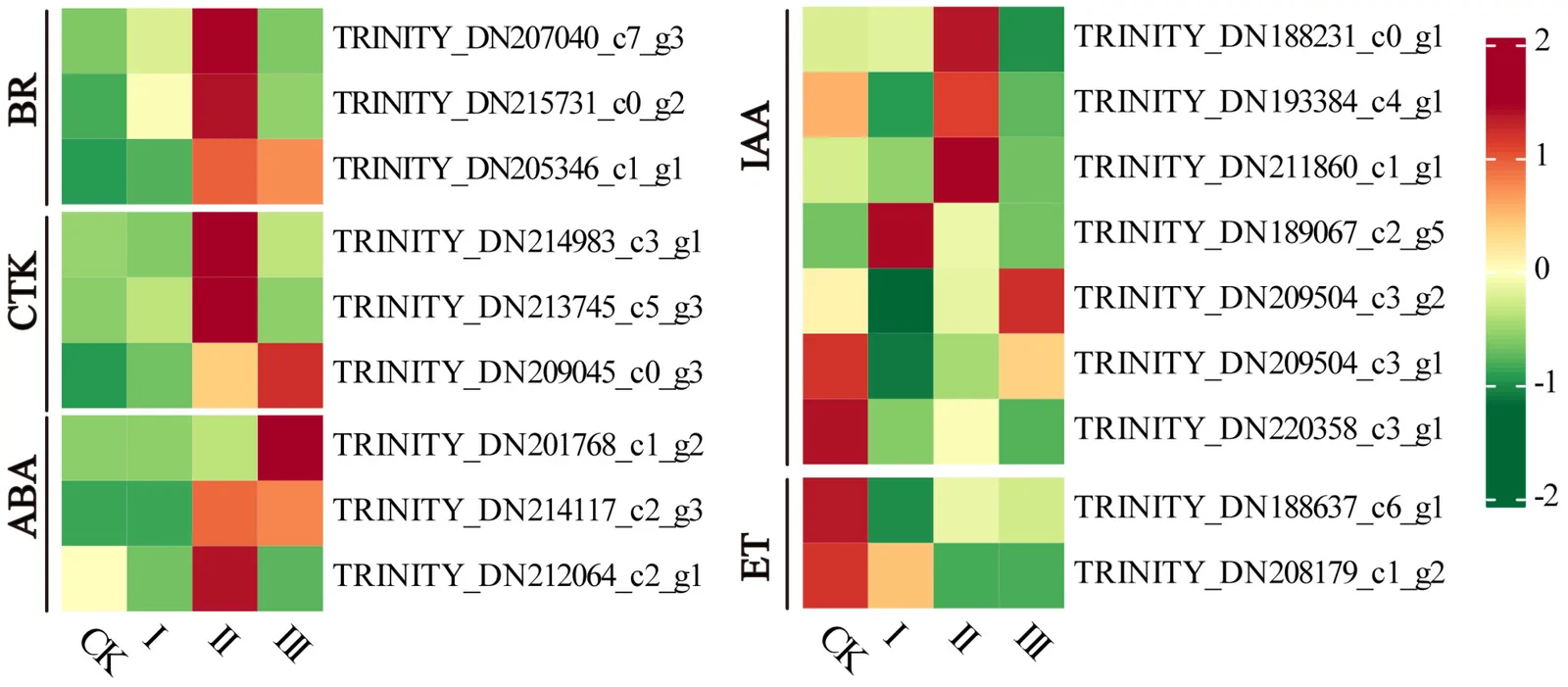
Heatmap showing expression patterns of plant hormone (BR, CTK, ABA)-related unigenes. Each column represents the mean expression value (log2 FPKM) of three biological replicates obtained from RNA-seq data.

The plant hormone signal transduction pathway was enriched with numerous shade-responsive DEGs, primarily involving three hormone modules: brassinosteroid (BR), cytokinin (CTK), and abscisic acid (ABA) (Figure 5). Unigenes in this pathway exhibited differentiated expression patterns across shading gradients. Transcriptomic data revealed an association between low light gradient and upregulated BR signaling gene set, as well as gradual downregulation of ABA pathway DEGs; coordinated transcriptional changes of multiple hormone modules may participate in seedling shade response. CTK pathway genes showed no uniform expression trend, participating in dynamic regulation of leaf development and cell division under shading. Auxin-related unigenes exhibited highly heterogeneous expression patterns across shading gradients without uniform up or downregulation. Two ethylene-associated transcripts also displayed divergent expression responses to different shading intensities. Similar to other hormone-related genes, these patterns represent transcript-level associations with shading treatments. The coordinated action of different hormone pathways reshapes plant morphology and balances stress resistance, representing a core regulatory mechanism for plant adaptation to low-light environments.

### 2.8. Phenylpropanoid Biosynthesis Pathway Under Shading Stress

**Figure 6:**
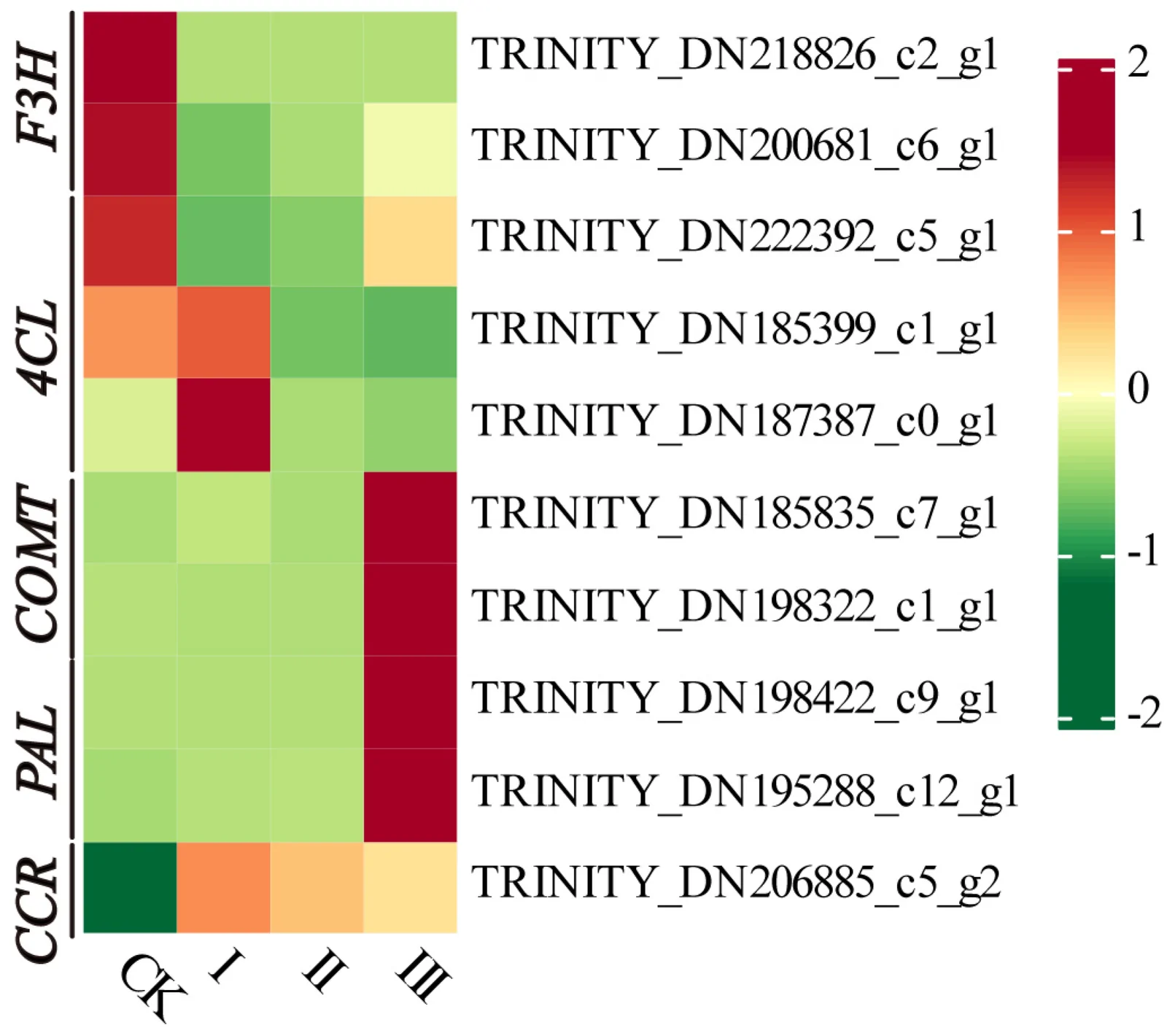
Heatmap showing expression profiles of unigenes involved in phenylpropanoid biosynthesis, with core pathway enzyme genes marked. Each column represents the mean expression value (log2 FPKM) of three biological replicates obtained from RNA-seq data.

The phenylpropanoid biosynthesis pathway, responsible for lignin, flavonoid, and phenolic antioxidant synthesis, was significantly enriched under shading treatments (Figure 6). Key rate-limiting genes in this pathway included phenylalanine ammonia-lyase (PAL), 4-coumarate-CoA ligase (4CL), cinnamoyl-CoA reductase (CCR), caffeic acid O-methyltransferase (COMT), and the flavonoid synthesis key gene F3H. Unigenes associated with lignin and flavonoid branches generally exhibited negative Z-scores under heavy shading, indicating that low light suppresses the expression of key enzyme genes in secondary metabolism. This suppression leads to reduced synthesis of photoprotective flavonoids and cell wall lignin, resulting in insufficient accumulation of light-shielding substances in leaves and decreased capacity for photooxidative stress resistance.

### 2.9. Carbon Metabolism Pathway Under Shading Stress

**Figure 7:**
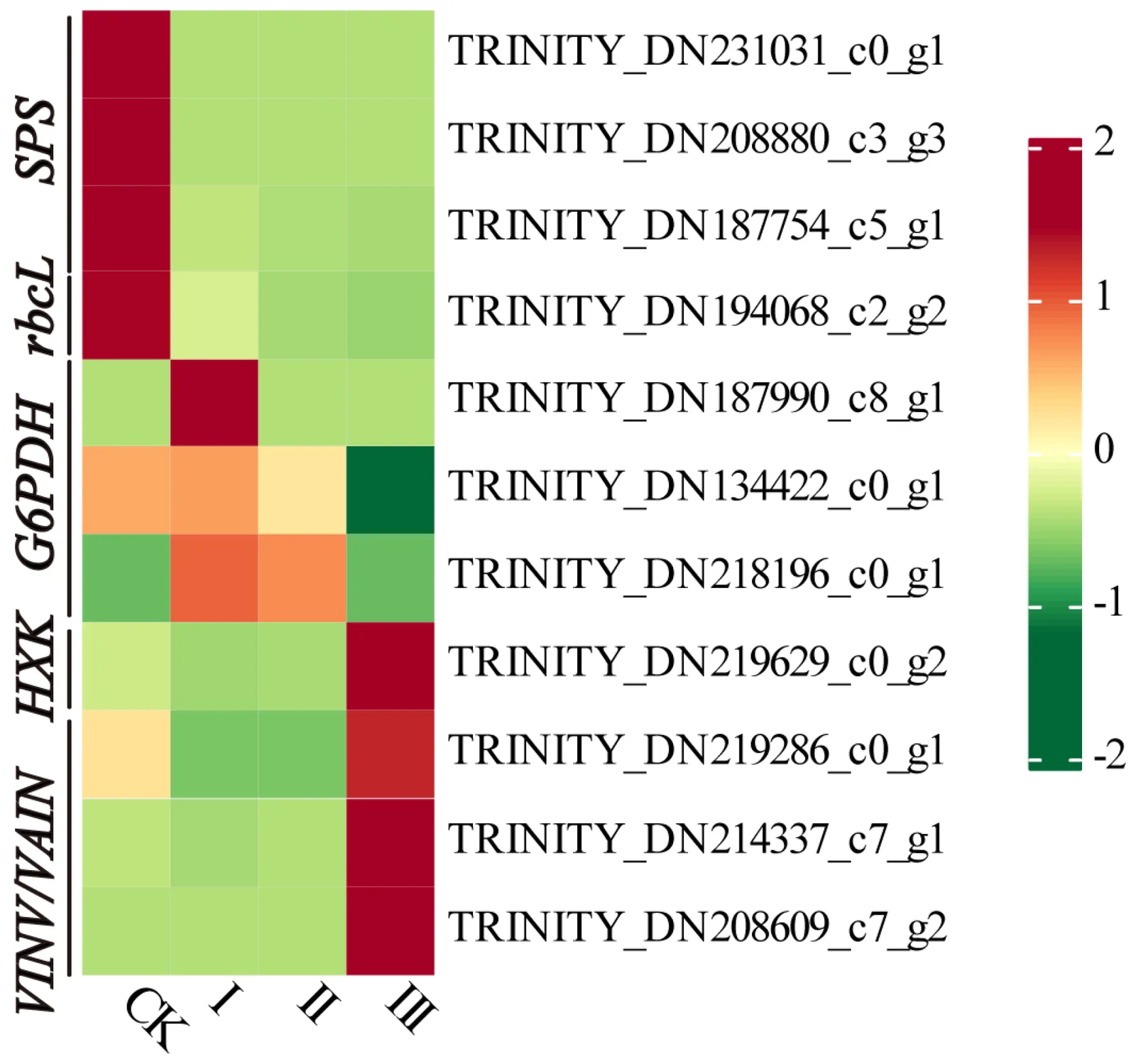
Heatmap illustrating expression patterns of unigenes participating in carbon metabolism, and core carbon metabolic functional genes are marked on the plot. Each column represents the mean expression value (log2 FPKM) of three biological replicates obtained from RNA-seq data.

Starch and sucrose carbon metabolism represented another core shade-responsive pathway (Figure 7). Key genes in the Calvin cycle, sucrose synthesis, and sugar catabolism were all significantly downregulated, including the Rubisco large subunit rbcL, sucrose phosphate synthase (SPS), vacuolar invertase (VINV/VAIN), hexokinase (HXK), and glucose-6-phosphate dehydrogenase (G6PDH). All unigenes in this pathway showed continuously decreasing expression with increasing shading intensity, with Z-scores becoming increasingly negative. Low light results in insufficient photosynthetic carbon fixation substrates, simultaneously inhibiting sucrose synthesis, transport, and storage processes. Overall carbon metabolic flux was blocked, ultimately leading to decreased biomass accumulation under shading.

### 2.10. The Validation of the Expression Levels of Genes Related to Different Pathways Using qRT-PCR

**Figure 8:**
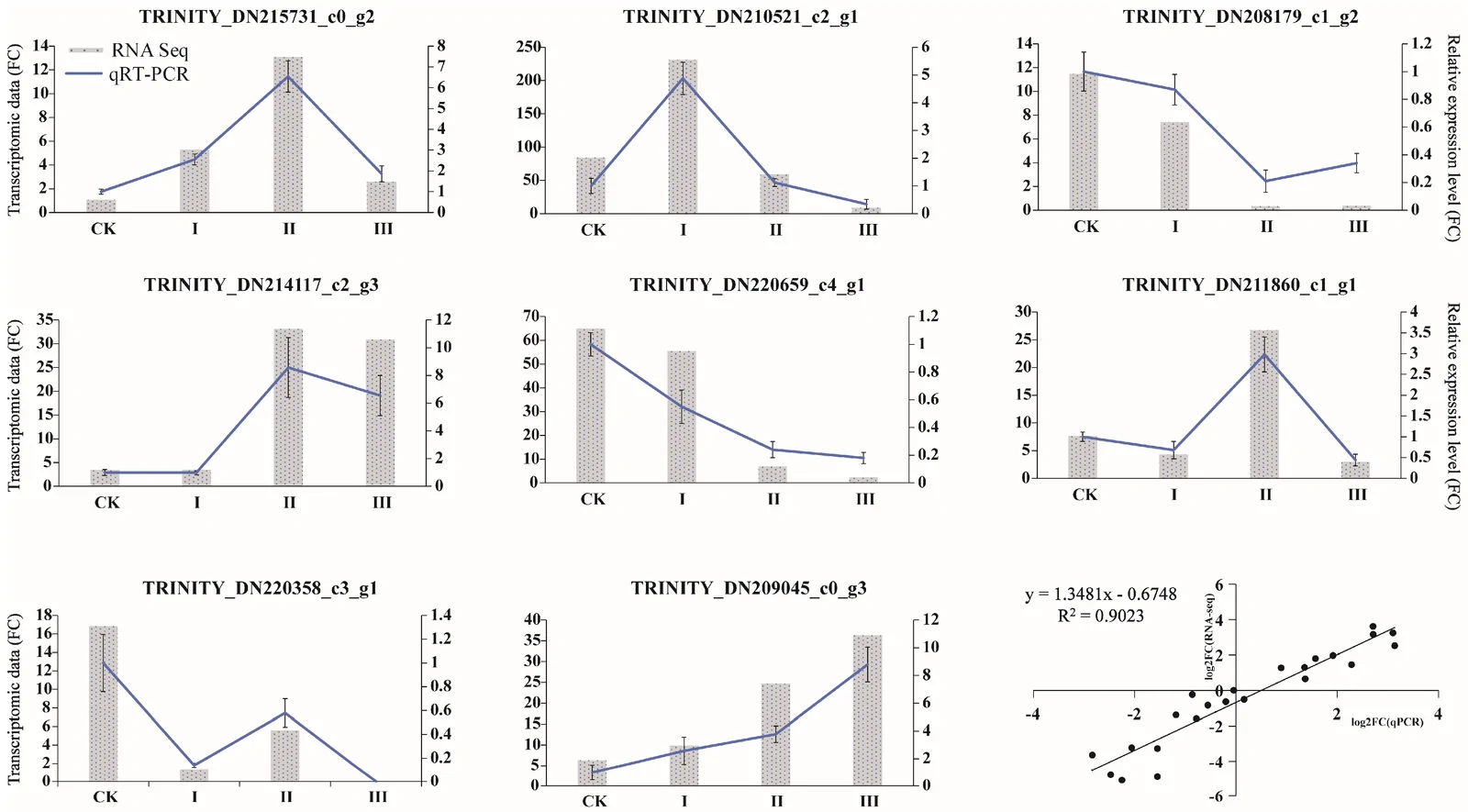
The RNA-seq data were validated by quantitative real-time polymerase chain reaction (qRT-PCR) validation. The bars represent the RNA-seq data, and the line graph shows the qRT-PCR data.

To verify the credibility of the transcriptional data, eight differentially expressed genes (DEGs) were randomly selected for quantitative real-time polymerase chain reaction (qRT-PCR) validation, including five upregulated genes and three downregulated genes. The relative expression levels of these genes between the shading treatment group and the control group were compared, and correlation analysis was conducted with the RNA-seq data. The results showed that the data obtained from qRT-PCR were consistent with the expression trends reflected by the RNA-seq data. The correlation coefficient between the shading treatment group and the control group was 0.9023. This indicates that the transcriptional data have extremely high reliability (Figure 8).

## 3. Discussion

### 3.1. Moderate Shading Promotes Growth While Heavy Shading Inhibits Development

Light serves as the energy source for all plant life activities and directly influences external morphological development. When light intensity changes, plants adapt to environmental variations by modifying morphology and adjusting biomass allocation [25]. In this study, *C. viminalis* seedlings exhibited optimal growth performance under 20% shading, with maximum plant height, basal diameter, and survival rate. As light intensity decreased further, growth parameters declined. This finding is consistent with previous studies on potato [26] and *Leymus chinensis* [27] under shading conditions.

Plants typically respond to shading stress by increasing plant height to capture more light for photosynthesis, ensuring normal growth [23]. However, when light intensity dropped to 15–20% (Treatment III), *C. viminalis* seedlings failed to increase height, and growth rates decreased or even resulted in mortality. This is likely because extremely low light intensity severely inhibits photosynthesis, preventing the acquisition of sufficient resources for normal growth. The unimodal response of growth parameters to shading intensity observed in this study suggests that *C. viminalis* seedlings have an optimal light range for growth, with both excessive and insufficient light being detrimental, which is consistent with previous studies [28].

The physiological basis for this growth pattern is reflected in the changes in photosynthetic parameters. Moderate shading (Treatment I) enhanced Pn, Gs, Ci, and Tr. The enhanced Pn under moderate 20% shading is hypothesized to be partially related to relieved high-light-induced photoinhibition; direct evidence from chlorophyll fluorescence parameters will be supplemented in future trials. Park [29] established six shading gradients and equipped them with complete fluorescence indicators, confirming that strong light causes damage to PSII. This species exhibits optimal growth under 35% shading. There is a difference in the suitable shading degree between the two species (20% vs. 35%). In contrast, heavy shading (Treatment III) significantly reduced all photosynthetic parameters, confirming severe photosynthetic limitation [30]. The accumulation of soluble proteins and soluble sugars under moderate shading suggests enhanced metabolic activity and osmoprotectant synthesis, while the increase in MDA and proline under heavy shading indicates oxidative stress and membrane damage [31,32].

A key limitation of the present experiment is the lack of continuous spectral and integral light quantity monitoring. Commercial plastic shade nets simultaneously reduce photosynthetic photon flux density and alter the R:FR ratio, a core signal triggering plant shade avoidance syndrome. Differences in seedling morphology, photosynthesis and transcriptional profiles among treatments were jointly shaped by combined changes in light attenuation, light spectral composition and minor microclimate variation introduced by multi-layer shade nets.

### 3.2. Photosynthesis Suppression and Hormonal Regulation Mediate Shade Responses

Transcriptomic analysis revealed that photosynthesis pathway genes were globally downregulated under shading stress, consistent with the observed decrease in photosynthetic rates. The suppression of light-harvesting complex genes (LHCA2, LHCB1), photosystem core genes (psbR), electron transport genes (PetE), and ATP synthase genes (ATPβ) indicates that shading reduces light energy capture, electron transfer, and ATP production at the molecular level. The downregulation of HemA, a key gene in chlorophyll biosynthesis, further explains the reduced chlorophyll content under shading [33].

The plant hormone signal transduction pathway exhibited complex regulatory patterns under shading [34]. The upregulation of brassinosteroid (BR) signaling genes is particularly noteworthy, as BRs are known to mediate shade avoidance responses, including stem elongation and hyponastic growth [35]. This is consistent with the observation that seedlings under moderate shading exhibited increased height (Figure 1). The downregulation of ABA pathway genes with increasing shading intensity suggests reduced stress defense activation under low light, which may explain the decreased accumulation of stress-related metabolites [36]. This study only analyzed transcriptional variation of hormone signal genes; endogenous BR/ABA hormone quantification, pathway protein activity and gene functional verification are required in follow-up work to confirm causal regulatory relationships.

### 3.3. Suppression of Phenylpropanoid and Carbon Metabolism Under Heavy Shading

Phenylpropanoid biosynthesis, responsible for lignin, flavonoid, and phenolic antioxidant synthesis, was significantly suppressed under heavy shading. The downregulation of key genes including *PAL*, *4CL*, *CCR*, *COMT*, and *F3H* indicates reduced production of photoprotective flavonoids and cell wall lignin [37]. Flavonoids serve as crucial non-enzymatic antioxidants that effectively neutralize excessive ROS [38], reducing oxidative damage and improving plant survival under environmental stress. The decreased expression of these genes under heavy shading suggests that plants under severe light limitation may have compromised antioxidant capacity, contributing to the observed increase in MDA content and reduced survival [27].

Carbohydrates are the primary energy source for plant cells and also function as critical signaling molecules that regulate plant responses to various stresses, including salinity, alkalinity, and shading [39]. Carbon metabolism genes, including those involved in the Calvin cycle (*rbcL*), sucrose synthesis (*SPS*), and sugar catabolism (*VINV*, *HXK*, *G6PDH*), were also downregulated under shading [40]. This global suppression of carbon metabolic flux indicates that low light limits photosynthetic carbon fixation, reducing substrate availability for sucrose synthesis, transport, and storage [41,42]. The resulting decrease in energy and carbon skeletons for growth and development explains the reduced biomass accumulation under heavy shading.

### 3.4. Integrated Model of Shade Response in C. viminalis

**Figure 9:**
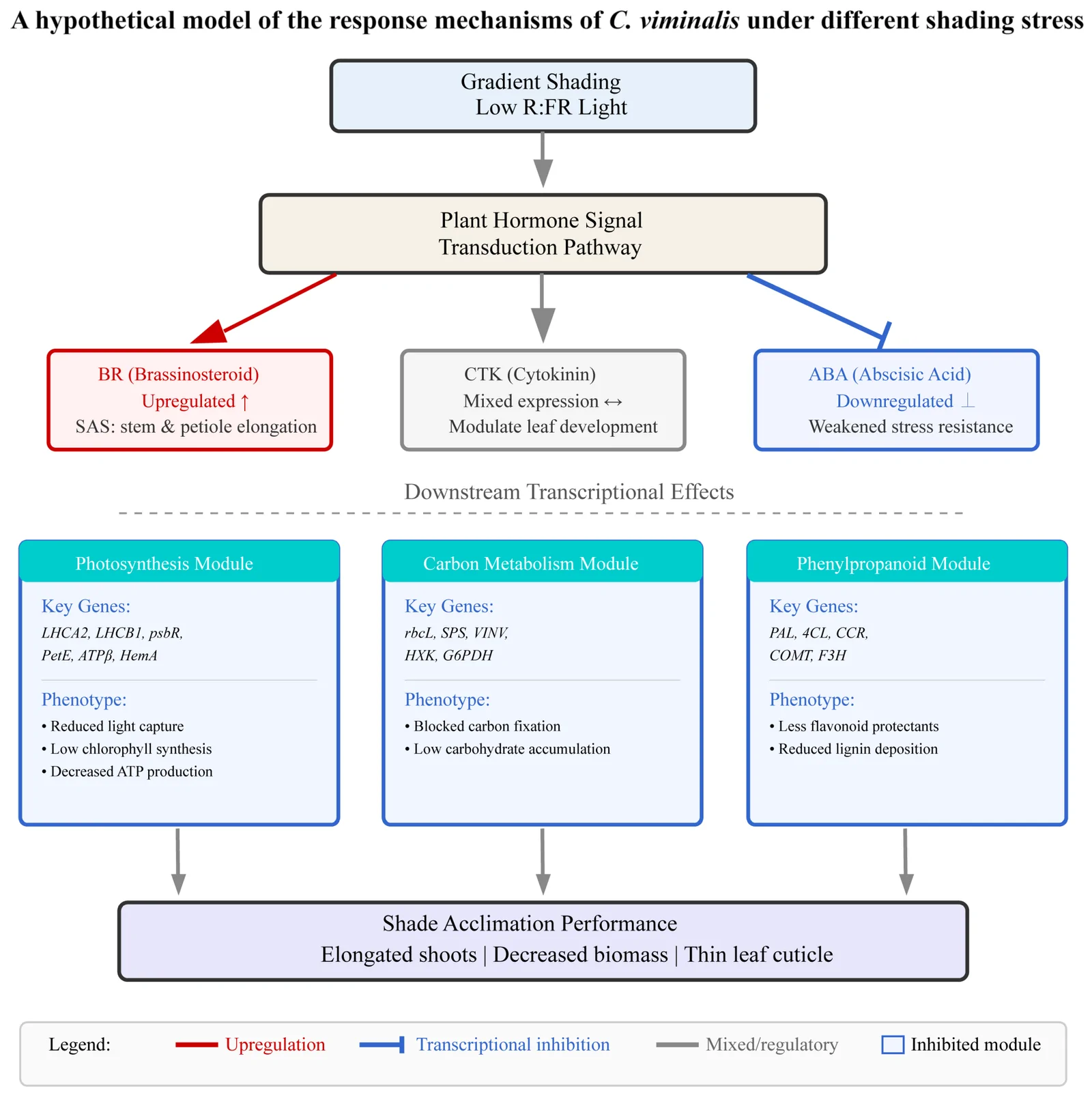
A hypothetical model of the response mechanisms of *C. viminalis* under different shading stress. This model is a hypothetical transcriptional regulatory framework integrated from morphological, physiological and RNA-seq data; dashed regulatory pathways require further biochemical and gene functional verification. The upward arrow indicates up-regulation, the arrows pointing left and right represent multiplex expression, and the vertical symbol represents down-regulation.

Based on our physiological and transcriptomic findings, we propose an integrated model for shade responses in *C. viminalis* seedlings (Figure 9). Under moderate shading (approximately 20% light reduction), plants maintain relatively high photosynthetic efficiency, with enhanced stomatal conductance and carbon fixation. BR signaling is activated to promote shade avoidance growth (height elongation), while secondary metabolism (phenylpropanoid and flavonoid biosynthesis) is moderately induced to provide antioxidant protection. This balanced response allows for optimal growth and survival. Under heavy shading (75% light reduction), photosynthesis is severely suppressed at the transcriptional level, with global downregulation of light-harvesting, electron transport, and carbon fixation genes. Carbon metabolism is compromised, reducing energy and substrate availability. Phenylpropanoid biosynthesis is suppressed, diminishing antioxidant capacity. ABA signaling is attenuated, further reducing stress defense. The cumulative effect of these molecular changes results in inhibited growth, increased oxidative damage, and reduced survival.

These findings highlight the importance of appropriate light management in *C. viminalis* cultivation. For nursery production, maintaining approximately 20% shading (80% relative light intensity) appears optimal for seedling growth and survival. This light level balances photosynthetic efficiency with protection against photoinhibition, while avoiding the detrimental effects of severe light limitation on carbon metabolism and secondary metabolism.

## 4. Materials and Methods

### 4.1. Plant Materials and Shading Treatments

One-year-old *Calamus viminalis* seedlings of uniform size (mean height: 15.12 cm; mean basal diameter: 3.34 mm) were used as experimental materials. Prior to treatment, all seedlings were acclimated for one month under uniform conditions. The experiment was conducted at the Yongmao Ecological Nursery Base in Sanya, Hainan Province, China (109°35′41″ E, 18°19′21″ N). The site has a tropical maritime monsoon climate with an average annual temperature of 25.4 °C, annual precipitation exceeding 1800 mm, and approximately 2563 h of annual sunshine.

Four shading treatments were established using shade nets with different densities: CK (0% shading, full sunlight), Treatment I (20% ± 3% shading, single-layer net), Treatment II (50% ± 3% shading, double-layer net), and Treatment III (75% ± 3% shading, triple-layer net). Each treatment consisted of 60 seedlings. Four independent shading enclosures were built for each treatment, 15 uniform seedlings per shelter, each shelter acting as an independent biological replicate at the enclosure level. All pots were randomly arranged inside each shelter. The four replicate shelters of each treatment were randomly distributed across the nursery field to homogenize background microclimate differences. Shade nets were constructed as 1.5 m high cubic enclosures, with the bottom raised 40 cm above the ground to ensure ventilation. This study only quantified the nominal shading attenuation percentage of each shade net via a handheld luxmeter (LX-1010b, HE YI INSTRUMENTS AND METERS, Shanghai, China) at midday on sunny days before trial initiation. Shade nets with different mesh densities simultaneously adjust light intensity and light spectral composition, both of which jointly drive seedling morphological and physiological responses.

### 4.2. Growth Parameter Measurements

After 360 days of treatment, plant height (measured from the soil surface to the highest fully expanded leaf base), basal diameter (measured 1 cm above the soil surface), leaf area (second leaf from the top), and survival rate were recorded for each treatment. Plant height was measured with a ruler (precision: 0.1 cm), basal diameter with a vernier caliper (precision: 0.01 mm), and leaf area with a leaf area meter (precision: 0.01 cm^2^). Survival rate was calculated as the percentage of surviving plants relative to the initial number. All 60 seedlings per treatment (4 independent shelters × 15 seedlings) were measured for height, basal diameter and leaf number; survival rate statistics adopted shelter-level population data.

### 4.3. Photosynthetic Parameter Measurements

Photosynthetic parameters were measured on clear mornings (9:00–11:00 AM) using a Li-6400 portable photosynthesis system (Li-COR, 4647 Superior Street, P.O. Box 4425, Lincoln, NE, USA). During all gas exchange measurements, the Li-6400 leaf cuvette was set to standardized constant conditions: PPFD (1200 μmol m^−2^s^−1^), CO_2_ concentration (400μmol mol^−1^), 28 °C, 65% relative humidity. The following parameters were recorded: net photosynthetic rate (Pn), transpiration rate (Tr), intercellular CO_2_ concentration (Ci), and stomatal conductance (Gs). Water use efficiency (WUE) was calculated as Pn/Tr, and the stomatal limitation value (Ls) was calculated as 1− Ci/Ca. Measurements were performed on fully expanded leaves with the leaf chamber temperature, CO_2_ concentration, and replaced it with standardized artificial cuvette environment control. Six fully expanded functional leaves from independent shelter replicates were measured per treatment (6 biological replicates), with 2 stable data recordings per leaf, and average values used for subsequent statistical analysis.

### 4.4. Physiological Parameter Measurements

Leaf samples were collected after photosynthetic measurements for physiological analysis. Malondialdehyde (MDA) content was determined using the thiobarbituric acid colorimetric method [43]. Superoxide dismutase (SOD) activity was measured using the nitroblue tetrazolium (NBT) photochemical reduction method [44]. Peroxidase (POD) activity was determined using the guaiacol method. Catalase (CAT) activity and proline (Pro) content were measured using standard protocols [45]. Soluble proteins were determined via the Coomassie brilliant blue G-250 colorimetric method, with bovine serum albumin as the standard curve reference, and absorbance measured at 595 nm [46]. Soluble sugars were determined using the anthrone–sulfuric acid colorimetric method, the glucose standard curve, and absorbance read at 625 nm [47]. Each physiological parameter was assessed with three biological replicates.

### 4.5. RNA Extraction, Library Construction, and Sequencing

Leaf sampling was conducted on the 360th day of shading treatment; the 2nd fully expanded functional leaf from the seedling top was collected; 3 independent biological replicates (from different shading shelters) per treatment, total 12 RNA-seq libraries. Total RNA was extracted by using a Trizol reagent kit (Invitrogen, Carlsbad, CA, USA) according to the manufacturer’s protocol. RNA quality was assessed on an Agilent 2100 Bioanalyzer (Agilent Technologies, Palo Alto, CA, USA) and checked via RNase-free agarose gel electrophoresis. After total RNA was extracted, eukaryotic mRNA was enriched with Oligo (dT) beads. The purified double-stranded cDNA fragments were end repaired, a base was added, and the fragments were ligated to Illumina sequencing adapters. The ligation reaction mixture was purified with AMPure XP Beads (1.0×). Ligated fragments were subjected to size selection via agarose gel electrophoresis and amplified via polymerase chain reaction (PCR). The resulting cDNA library was sequenced via Illumina NovaSeq 6000 by Berry Genomics Co. (Beijing, China).

### 4.6. Transcriptome Data Analysis

Raw sequencing reads were filtered to remove low-quality reads and adapter sequences using fastp. Clean reads were then assembled de novo using Trinity to generate a reference transcriptome for *Calamus viminalis*. For expression quantification, clean reads were mapped back to the assembled transcriptome using Bowtie2(v2.5.5), and transcript abundances were estimated as transcripts per million (TPM) using RSEM(v1.3.3). Differential expression analysis was performed using DESeq2v1.42() with thresholds of |log_2_(fold change)| ≥ 1 and adjusted *p*-value < 0.05.

### 4.7. GO and KEGG Enrichment Analysis

Gene Ontology (GO) functional enrichment analysis was performed using the clusterProfiler R package(R 4.6.1). Kyoto Encyclopedia of Genes and Genomes (KEGG) pathway enrichment analysis was conducted using the same package, with a significance threshold of *p* < 0.05. Heatmaps were generated using the pheatmap R package with Ilog2FoldChangeI > 1 and FDR < 0.05 normalization.

### 4.8. Statistical Analysis

All data are presented as the mean ± standard deviation (SD). Statistical analyses were performed using SPSS 13.0 software. One-way analysis of variance (ANOVA) followed by Duncan’s multiple range test was used to determine significant differences among treatments. Differences were considered significant at *p* < 0.05.

### 4.9. qRT-PCR

qRT-PCR analysis was performed with SYBR Premix Ex TaqTM (TaKaRa, Dalian, China). ACTIN was used as the internal control for target genes to standardize the RNA quantity for evaluating relative expression levels. The relative expression level was calculated via the 2^-ΔΔCt^ method [48]. All of the primers are listed in Table S3. At least three technical replicates were carried out for each sample and three independent biological replicates were performed.

## 5. Conclusions

This study employed a combined approach of physiology and transcriptomics to investigate the key regulatory pathways and adaptive mechanisms of *C. viminalis* under different shading treatments. Through transcriptomics analysis, four regulatory pathways related to shading stress were identified. Overall, shading stress inhibits genes related to photosynthesis. Genes related to the BR signaling pathway are upregulated to mediate the shading avoidance response, while ABA pathway genes are downregulated. Under intense shading conditions, phenylpropanoid biosynthesis genes and carbon metabolism-related genes are significantly inhibited. This has been consistently verified at the plant phenotypic and physiological levels. These findings provide theoretical basis for *Calamus viminalis* seedling nursery management under controlled nursery conditions. Future controlled experiments deploying LED light systems will be used to independently separate light intensity and light quality effects, to disentangle their respective regulatory roles in *C. viminalis* seedling shade adaptation. Future research should focus on verifying the functions of the key candidate genes identified in this study and exploring their shading responses under field conditions.

## Data Availability

The original contributions presented in this study are included in the article/Supplementary Materials; further inquiries can be directed to the corresponding author.

## Supplementary Materials

The following supporting information can be downloaded at: https://www.mdpi.com/article/10.3390/ijms27177804/s1.

## References

[1] Lin W.K. (2015). Resources and Distribution of Rattan in China. Plant Sci. J..

[2] Manohara T.N. (2013). Nutritional Evaluation of Shoots of Two Rattans of Northeast India—*Calamus flagellum* Griff. ex Mart. and *C. floribundus* Griff. (Arecaceae)1. Econ. Bot..

[3] Li R., Xu H., Yang J., Yin G. (2021). A Review of Relationship Between Rattan and Water. For. Stud. China.

[4] Dong S.F., Lu J., Peng Y.W. (2015). Effect of Shading and Fertilization on Seedling Growth of *Calamus gracilis*. J. West China For. Sci..

[5] Chen B., Li Y., Liu G., Fan S., Yang H., Su H. (2020). Effects of the interaction between shade and drought on physiological characteristics in *Calamus viminalis* seedlings. Not. Bot. Horti Agrobot. Cluj-Napoca.

[6] Bertamini M., Muthuchelian K., Rubinigg M., Zorer R., Velasco R., Nedunchezhian N. (2006). Low-night temperature increased the photoinhibition of photosynthesis in grapevine (*Vitis vinifera* L. cv. Riesling) leaves. Environ. Exp. Bot..

[7] Dai Y., Shen Z., Liu Y., Wang L., Hannaway D., Lu H. (2009). Effects of shade treatments on the photosynthetic capacity, chlorophyll fluorescence, and chlorophyll content of *Tetrastigma hemsleyanum* Diels et Gilg. Environ. Exp. Bot..

[8] Paradiso R., Proietti S. (2021). Light-Quality Manipulation to Control Plant Growth and Photomorphogenesis in Greenhouse Horticulture: The State of the Art and the Opportunities of Modern LED Systems. J. Plant Growth Regul..

[9] Bialevich V., Zachleder V., Bišová K. (2022). The Effect of Variable Light Source and Light Intensity on the Growth of Three Algal Species. Cells.

[10] Bueno P.M.C., Vendrame W.A. (2024). Wavelength and Light Intensity Affect Macro- and Micronutrient Uptake, Stomata Number, and Plant Morphology of Common Bean (*Phaseolus vulgaris* L.). Plants.

[11] Huang C.J., Wei G., Jie Y.C., Xu J.J., Anjum S.A., Tanveer M. (2016). Effect of shade on plant traits, gas exchange and chlorophyll content in four ramie cultivars. Photosynthetica.

[12] Gehring C.A. (2003). Growth responses to arbuscular mycorrhizae by rain forest seedlings vary with light intensity and tree species. Plant Ecol..

[13] Zhang T., Yan Q., Yuan J., Zhang J. (2022). Application of fertilization in changing light adaptability and improving growth of *Aralia elata* (Miq.) Seem. seedlings under various light conditions in temperate forests. J. Plant Physiol..

[14] Li S., Du J. (2025). Transcriptome analysis reveals novel insights into the mechanisms of *Davidia involucrata* Baill. response to high-light stress. Sci. Rep..

[15] Sun Y., Wang Y., Cao Q., Zhao Y., Xu X., Shi Y., Zhang L. (2025). Dynamic transcriptomic and metabolomic responses of rice under high light stress. Plant Sci..

[16] Yin G., Xu H., Zhang W. (1988). A preliminary study on the effect of different levels of light intensity on the growth of rattan seedlings. For. Res..

[17] Wang H., Seiler C., Sreenivasulu N., von Wirén N., Kuhlmann M. (2021). INTERMEDIUM-C mediates the shade-induced bud growth arrest in barley. J. Exp. Bot..

[18] Crocco C.D., Locascio A., Escudero C.M., Alabadí D., Blázquez M.A., Botto J.F. (2015). The transcriptional regulator BBX24 impairs DELLA activity to promote shade avoidance in *Arabidopsis thaliana*. Nat. Commun..

[19] Llorente B., D’Andrea L., Ruiz-Sola M.A., Botterweg E., Pulido P., Andilla J., Loza-Alvarez P., Rodriguez-Concepcion M. (2015). Tomato fruit carotenoid biosynthesis is adjusted to actual ripening progression by a light-dependent mechanism. Plant J..

[20] Du J., Jiang H., Sun X., Li Y., Liu Y., Sun M., Fan Z., Cao Q., Feng L., Shang J. (2018). Auxin and Gibberellins Are Required for the Receptor-Like Kinase ERECTA Regulated Hypocotyl Elongation in Shade Avoidance in *Arabidopsis*. Front. Plant Sci..

[21] Lu H., Lu C., Huang S., Liu W., Wang L., Yang C., Wang E., Li L. (2025). Rhizosphere microbes mitigate the shade avoidance responses in *Arabidopsis*. Cell Host Microbe.

[22] Liu D., Cui Y., Zhao Z., Zhang J., Li S., Liu Z. (2022). Transcriptome analysis and mining of genes related to shade tolerance in foxtail millet (*Setaria italica* (L.) P. Beauv.). R. Soc. Open Sci..

[23] Zhao Y., Zhou J., Xing D. (2014). Phytochrome B-mediated activation of lipoxygenase modulates an excess red light-induced defence response in *Arabidopsis*. J. Exp. Bot..

[24] Zhang F., Zhou R., Yuan R., Zhu X., Zhang X., Lamlom S.F., Shi D., Abdelghany A.M., Du J., Ren H. (2025). Transcriptome analysis of shade-induced growth and photosynthetic responses in soybean cultivars. PLoS ONE.

[25] Tang G.-L., Li X.-Y., Lin L.-S., Li L., Lu J.-R. (2013). Change of different shading on moisture conditions and the physiological response in *Alhagi sparsifolia*. Chin. J. Plant Ecol..

[26] Liu J., Tang X., Zhang H., Wei M., Zhang N., Si H. (2024). Transcriptome Analysis of Potato Leaves under Oxidative Stress. Int. J. Mol. Sci..

[27] Li X., Yu Q., Yao Z., Li S., Ma L., Su K., Yang G. (2025). The Combination of Physiological and Transcriptomic Approaches Reveals New Insights into the Molecular Mechanisms of *Leymus chinensis* Growth Under Different Shading Intensities. Int. J. Mol. Sci..

[28] Liu Q., Huang Z., Zou X., Ma X., Liu B. (2025). Adaptation strategies of *Cunninghamia lanceolata* seedlings to light intensity gradients based on morpho-physiological trade-offs. Front. Plant Sci..

[29] Park S.H., Lee J.H., Nam S.Y. (2023). An Analysis of the Growth and Photosynthetic Responses of Potted *Veronica pusanensis* Y.N.Lee according to the Shading Levels. J. People Plants Environ..

[30] Naseer M.A., Hussain S., Mukhtar A., Rui Q., Ru G., Ahmad H., Zhang Z.Q., Shi L.B., Asad M.S., Chen X. (2024). Chlorophyll fluorescence, physiology, and yield of winter wheat under different irrigation and shade durations during the grain-filling stage. Front. Plant Sci..

[31] Xue T., Zhang H., Zhang Y., Wei S., Chao Q., Zhu Y., Teng J., Zhang A., Sheng W., Duan Y. (2019). Full-length transcriptome analysis of shade-induced promotion of tuber production in *Pinellia ternata*. BMC Plant Biol..

[32] Di P., Yang X., Wan M., Han M., Zhang Y., Yang L. (2023). Integrative metabolomic and transcriptomic reveals potential mechanism for promotion of ginsenoside synthesis in Panax ginseng leaves under different light intensities. Front. Bioeng. Biotechnol..

[33] Ji S., Wang P., Grimm B. (2025). Modification of aggregation-prone regions of Arabidopsis glutamyl-tRNA reductase leads to increased stability while maintaining enzyme activity. Front. Plant Sci..

[34] Chen T., Zhang H., Zeng R., Wang X., Huang L., Wang L., Wang X., Zhang L. (2020). Shade Effects on Peanut Yield Associate with Physiological and Expressional Regulation on Photosynthesis and Sucrose Metabolism. Int. J. Mol. Sci..

[35] Li L., Wonder J., Helming T., van Asselt G., Pantazopoulou C.K., van de Kaa Y., Kohlen W., Pierik R., Kajala K. (2024). Evaluation of the roles of brassinosteroid, gibberellin and auxin for tomato internode elongation in response to low red: Far-red light. Physiol. Plant..

[36] Huang Y., Mei G., Zhu K., Ruan X., Wu H., Cao D. (2024). Shading treatment during late stage of seed development promotes subsequent seed germination and seedlings establishment in sunflower. Plant Sci..

[37] Jaakola L. (2013). New insights into the regulation of anthocyanin biosynthesis in fruits. Trends Plant Sci..

[38] Agati G., Tattini M. (2010). Multiple functional roles of flavonoids in photoprotection. New Phytol..

[39] Domingos S., Fino J., Cardoso V., Sánchez C., Ramalho J.C., Larcher R., Paulo O.S., Oliveira C.M., Goulao L.F. (2016). Shared and divergent pathways for flower abscission are triggered by gibberellic acid and carbon starvation in seedless *Vitis vinifera* L. BMC Plant Biol..

[40] Liu W., Luo R., Wang H., Jing Y., Zhao H., Zou W., Hou M., Song L. (2025). Integrated transcriptome and metabolome analyses reveal regulation mechanism of biomass and non-structural carbohydrate allocation in *Emmenopterys henryi* Oliv. under shade. Physiol. Mol. Biol. Plants.

[41] Xu W., Li P., Pu T., Liu Q., Han H., Li Y., Wu Z. (2025). Melatonin application alleviates adverse effects of low light on tobacco seedlings via enhancing antioxidant and carbohydrate metabolism. Front. Plant Sci..

[42] Wu W., Chen L., Liang R., Huang S., Li X., Huang B., Luo H., Zhang M., Wang X., Zhu H. (2025). The role of light in regulating plant growth, development and sugar metabolism: A review. Front. Plant Sci..

[43] Kong Q., Lin S., Wang R., Chen S., Ge K., Chen D. (2025). Changes in quality, endogenous enzyme activities, and their relationships during post-harvest storage of *Phlebopus portentosus*- an edible fungus. Food Chem..

[44] Zhang S., Bi Y., Tan H. (2025). Dark septate endophytic fungus *Alternaria* sp. 17463 regulates various antioxidant enzymes and compounds to mitigate salt stress caused by different anion salts. J. Biotechnol..

[45] Diao Q., Song Y., Qi H. (2015). Exogenous spermidine enhances chilling tolerance of tomato (*Solanum lycopersicum* L.) seedlings via involvement in polyamines metabolism and physiological parameter levels. Acta Physiol. Plant..

[46] Rekowski A., Langenkämper G., Dier M., Wimmer M.A., Scherf K.A., Zörb C. (2021). Determination of soluble wheat protein fractions using the Bradford assay. Cereal Chem..

[47] Wang X., Wang J., Zhu Y., Qu Z., Liu X., Wang P., Meng Q. (2024). Improving resilience to high temperature in drought: Water replenishment enhances sucrose and amino acid metabolisms in maize grain. Plant J..

[48] Pfaffl M.W. (2001). A new mathematical model for relative quantification in real-time RT-PCR. Nucleic Acids Res..

