# Supplementary material for: Physiological and Transcriptomic Analysis of Shading Stress Responses in Calamus viminalis Seedlings
Source: Int J Mol Sci. 2026 Aug 31;27(17):7804. doi: 10.3390/ijms27177804 (PMC13566859; doi:10.3390/ijms27177804)
Supplement: Supplementary file 1 [file ijms-27-07804-s001.zip › Suplyment FigureS1.pdf]

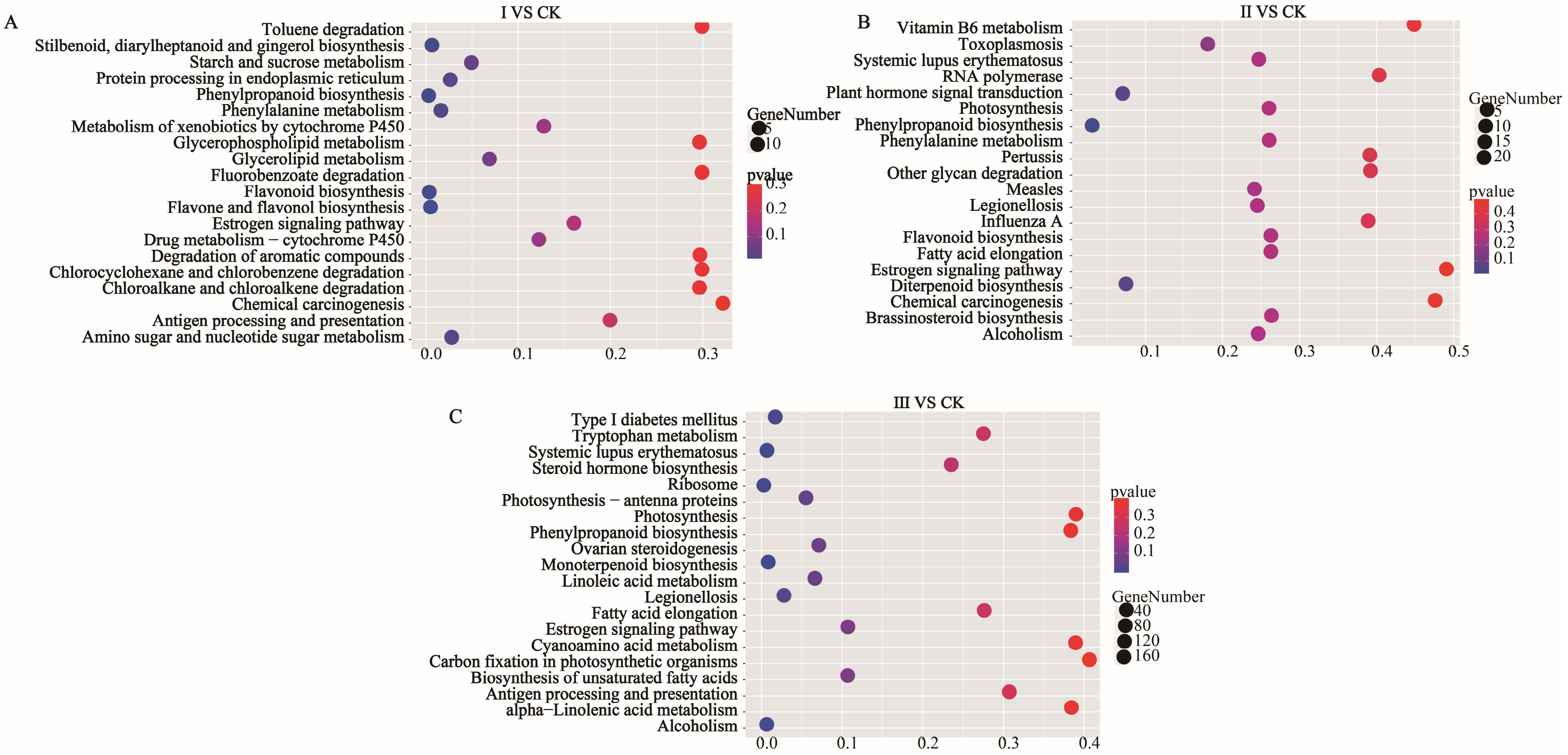

Figure S1. KEGG pathway enrichment analysis of DEGs in different shading treatments compared to control. (A) Treatment I vs. CK; (B) Treatment II vs. CK; (C) Treatment III vs. CK. The size of each dot represents the number of DEGs, and the color indicates the *p*-value.
